# Unmanned aerial platform-based multi-spectral imaging for field phenotyping of maize

**DOI:** 10.1186/s13007-015-0078-2

**Published:** 2015-06-24

**Authors:** M Zaman-Allah, O Vergara, J L Araus, A Tarekegne, C Magorokosho, P J Zarco-Tejada, A Hornero, A Hernández Albà, B Das, P Craufurd, M Olsen, B M Prasanna, J Cairns

**Affiliations:** International Maize and Wheat Improvement Center (CIMMYT), PO Box MP163, Peg Mazowe Rd, Mt Pleasant, Harare, Zimbabwe; Plant Physiology Unit, Department of Plant Biology, University of Barcelona, 08028 Barcelona, Spain; Laboratory for Research Methods in Quantitative Remote Sensing (Quantalab IAS-CSIC), Cordoba, Spain; Airelectronics, 28223 Pozuelo de Alarcón Madrid, Spain; International Maize and Wheat Improvement Center (CIMMYT), PO Box 1041, Nairobi, Kenya

**Keywords:** Maize, Phenotyping platform, Remote sensing, UAP, Nitrogen fertilization

## Abstract

**Background:**

Recent developments in unmanned aerial platforms (UAP) have provided research opportunities in assessing land allocation and crop physiological traits, including response to abiotic and biotic stresses. UAP-based remote sensing can be used to rapidly and cost-effectively phenotype large numbers of plots and field trials in a dynamic way using time series. This is anticipated to have tremendous implications for progress in crop genetic improvement.

**Results:**

We present the use of a UAP equipped with sensors for multispectral imaging in spatial field variability assessment and phenotyping for low-nitrogen (low-N) stress tolerance in maize. Multispectral aerial images were used to (1) characterize experimental fields for spatial soil-nitrogen variability and (2) derive indices for crop performance under low-N stress. Overall, results showed that the aerial platform enables to effectively characterize spatial field variation and assess crop performance under low-N stress. The Normalized Difference Vegetation Index (NDVI) data derived from spectral imaging presented a strong correlation with ground-measured NDVI, crop senescence index and grain yield.

**Conclusion:**

This work suggests that the aerial sensing platform designed for phenotyping studies has the potential to effectively assist in crop genetic improvement against abiotic stresses like low-N provided that sensors have enough resolution for plot level data collection. Limitations and future potential uses are also discussed.

## Background

To ensure improved agricultural productivity, the development and deployment of phenotyping technologies that enable monitoring of phenotypic changes of crop plants in the field is a critical component [1, 2]. Satellite imaging technologies have become an extremely useful tool for collecting data useful for various agricultural applications. However, the major challenges that limit their application in the area of crop improvement are the high cost and the lack of resolution for plot level crop data collection as well as the large revisit periods. The use of manned airborne remote sensing has demonstrated capabilities for large scale crop condition monitoring or for example yield and quality forecasting due to the high spatial and spectral resolutions of the sensors mounted. However in the case of breeding and except for big seed companies its high operating costs and the operational complexity involved have usually limited its use so far to research activities [3]. Unmanned aerial vehicle platforms (UAPs) equipped with sensors are emerging as an important, albeit affordable, component of precision agriculture and crop improvement [4]. The use of these platforms is becoming critical in crop phenotyping because of their ability to rapidly phenotype large numbers of plots and field trials in a dynamic way that can assist the identification and definition of the genetics behind crop yield variability. In addition, the traditional methods currently used, like visual senescence and plant vigor scorings deliver ranking that are variable, depending on the training and subjective appreciations of the staff devoted to that task. With optimum spatial and spectral resolutions, remote sensing from satellite and conventional aerial platforms can provide spatially and spectrally derived parameters for various purposes including crop condition [5–7], crop forecasting and yield predictions [8–10], disease detection and nutrient deficiency [11–13], and photosynthetic pigment content [14–16]. This becomes extremely important with regard to the increasing demand to support and accelerate progress in breeding for novel traits which at the same time requires to accurately measure increasingly large numbers of plants. With improvements in spatial, spectral and temporal resolution of aerial remote sensing, UAPs will enable near real-time visual assessment for crop monitoring in the field yield predictions, crop status mapping, weed detection, and disease and nutrient deficiency detection. Moreover the development of these miniaturized, affordable light-weight unmanned aerial platforms, with better flight control, have enabled the acquisition of high resolution images for various remote sensing applications. Preliminary reports on identification of damaged leaves using the normalized difference vegetation index (NDVI) showed a good similarity between the NDVI values as predicted by remote sensing using UAPs with that of ground truth [17]. Similarly, good potential from multispectral imaging sensors mounted on UAV platforms for physiological condition assessment [18, 19] and stress detection in different crops was reported [3] including hyperspectral imaging. In addition, these studies reported a good relationship between the predicted and validated values of leaf area index (LAI) in maize (r^2^ = 0.5) plants as well as chlorophyll concentration at the crop level.

Spectral measurements enable to derive a number of reflectance vegetation indices which have been introduced in both field research and breeding programs for large-scale phenotyping and dynamic estimation of biomass, greenness, nitrogen content, pigment composition, photosynthetic status, and water content [20, 21]. However so far their use in plant phenotyping under field conditions remains far more novel than their implementation under controlled (e.g. greenhouse or growth chamber) conditions [22].

Another critical area where aerial remote sensing can be useful is the characterization of spatial field variability which results usually from crop management history, spatial changes in soil characteristics and elevation gradients affecting water and nutrients movement. Spatial variability is a serious limitation to breeding efficiency because it creates variation of the stress level imposed within trials, which decreases the heritability of the phenotypic traits evaluated [23] and obstruct the detection of the genetic signal [24]. Spatial variability in crop productivity is even more evident when differences in resources such as soil N become limiting [25]. In low-input management systems Verhulst et al. [26] showed that standard deviation and coefficient of variation of NDVI values were high. Recently, Cairns et al. [27] have reported a very large residual and genotype × trial variances in the drought and combined drought and heat treatments relative to the well-watered treatment which resulted in reduced heritability for means estimates from the stressed trials. In addition, a combined analysis of the southern Africa regional trials of CIMMYT and partners also pointed out plot residual variance to be much higher under managed stress relative to nonstress trials [28]. These results highlight the need for measures to reduce the effects of field variability so as to increase the genetic signal to noise ratio. One way of addressing this spatial field variability problem is to collect soil information; but this has proven to be a laborious process. Therefore, aerial spectral imaging could be a quick and low-cost method for experimental field characterization.

To be relevant for breeding, plant phenotyping, should allow to objectively select key trait(s) under the least spatial field variability conditions. This underlines the critical need to use the “right” tools for data collection and for minimizing the spatial variability. To date, only few studies have reported attempts to use UAP’s remote sensing for spatial field variation assessment and crop phenotyping in the field. This work reports a proof of concept exercise on how a UAV-based remote sensing platform equipped with sensor for multispectral imaging could be used for experimental field spatial variability and maize phenotyping in the field under low-nitrogen stress conditions.

## Results and discussion

### Experimental field characterization

**Figure 1:**
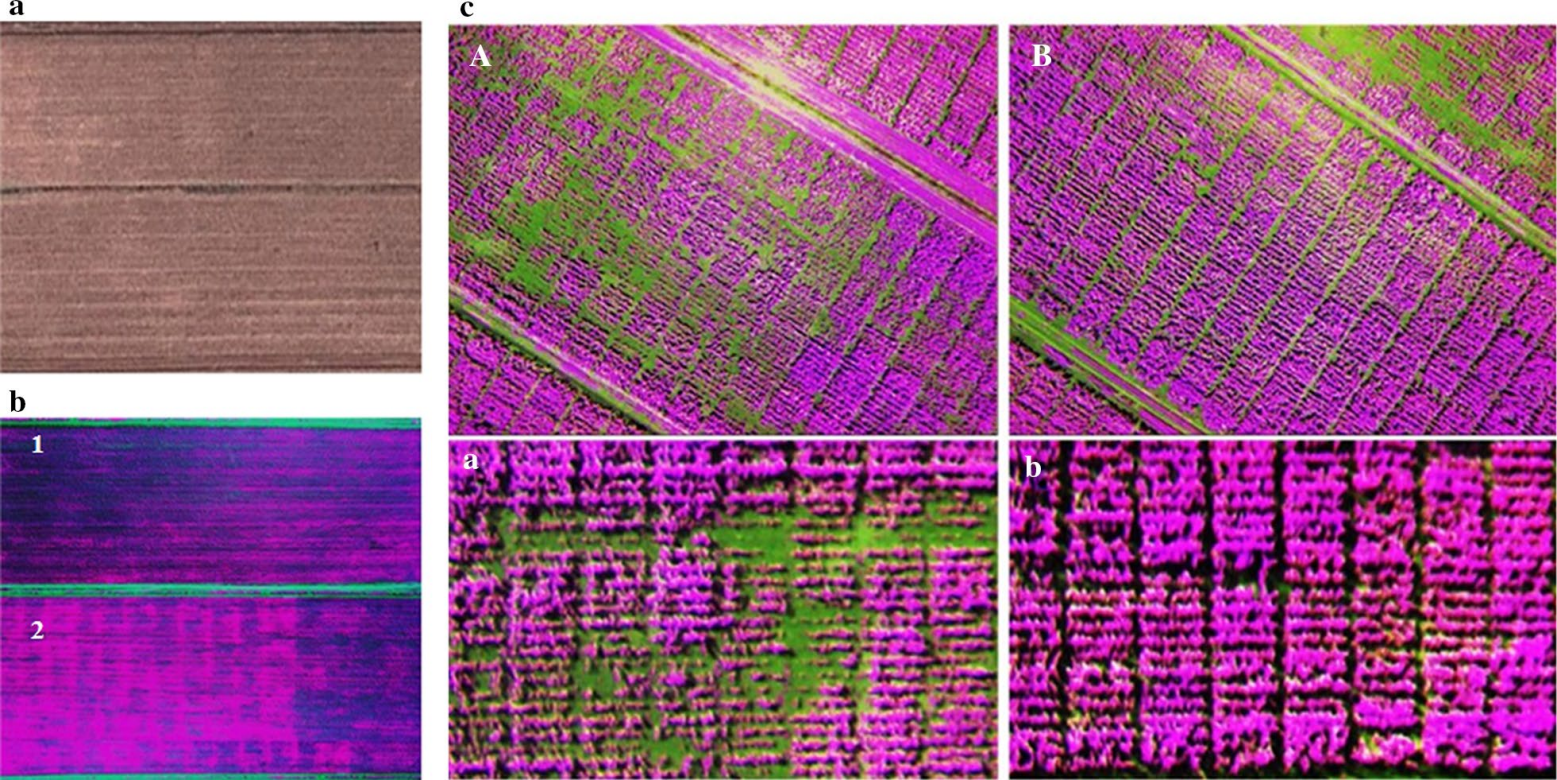
**a** Satellite view (Googlemap) of low-N fields at the CIMMYT Harare station, **b** Spectral reflectance of wheat plants grown at high density on the low-N fields prior to booting. (*filled purple square* and *filled pink square* indicate very low nitrogen and relatively higher nitrogen levels, respectively). The fields were depleted of soil N for (1) 5 years and (2) 4 years by growing maize without any N application. **c** Multispectral images showing management related field variability of low-N fields at the CIMMYT Harare station planted with maize trials. **A**, **a** High variation in a poorly managed field and **B**, **b** well-managed field.

**Figure 2:**
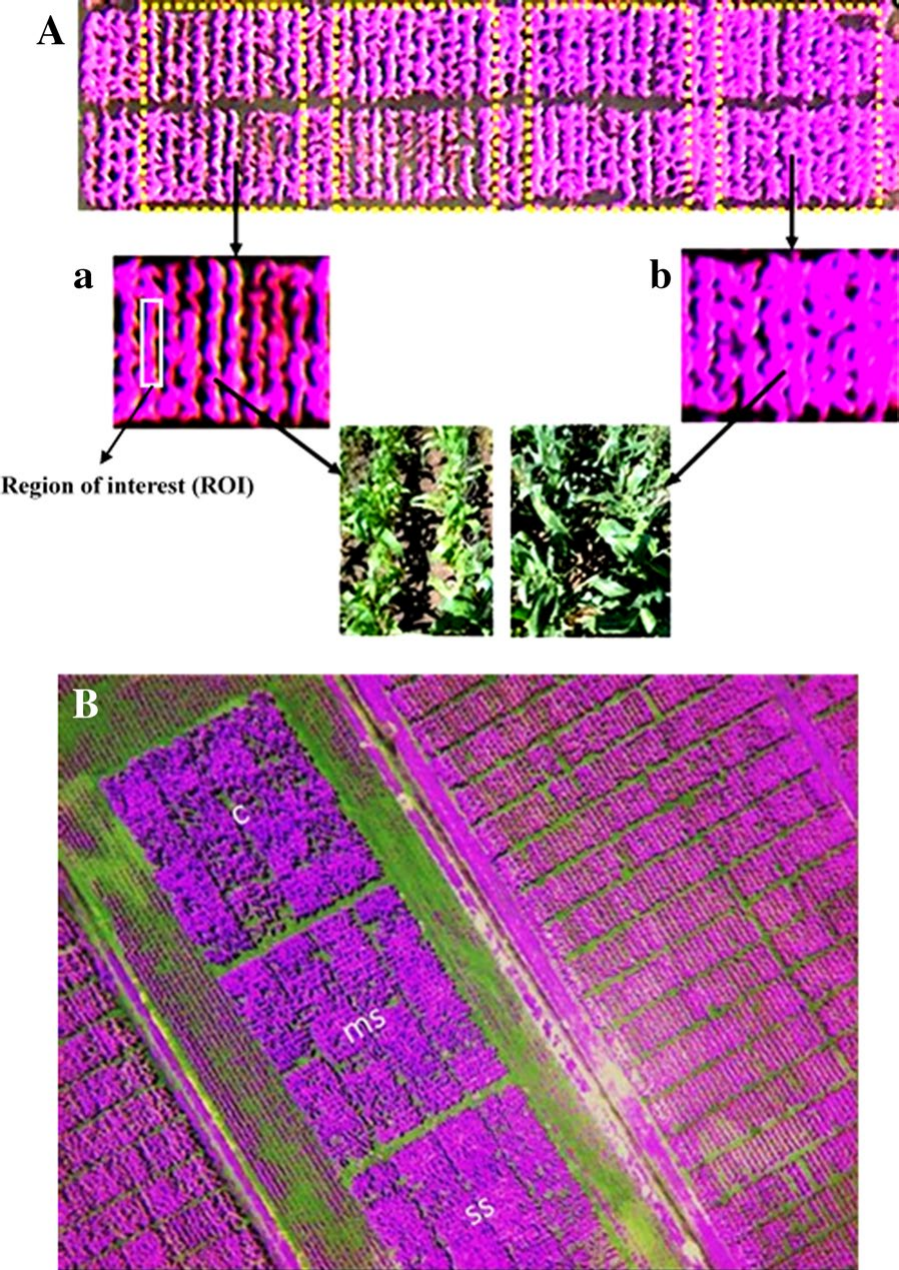
**A** Multispectral images of plots under different N-application rates. *a* N-stressed plot and *b* Non-stressed plot. **B** Maize plants grown under severe N-stress (*SS*), mild N-stress (*ms*) and optimum N supply (*C*).

Experimental fields can be characterized using crop management history, spatial changes in soil characteristics, elevation gradients affecting water and nutrients movement. Spatial variability in crop productivity is usually more evident when differences in resources such as soil N become limiting [25]. In low-input management systems Verhulst et al. [26] showed that standard deviation and coefficient of variation of NDVI values were high. This has negative implications for the quality of data to be collected and ultimately the selection efficiency in crop improvement. To address this major constraint, collecting soil information is crucial as it can help understanding grain yield variation, but this has proven to be a laborious process. In this work, we have tested aerial spectral imaging as a quick and low-cost tool for experimental field characterization. The images taken with the UAP showed differences in uniformity related to N-availability in the managed low-N fields (Figure 1a). Field 2 was the most heterogeneous with large number of plants rows showing high NDVI values and higher standard deviation and CV than field 1 (Table 1). This was the result of the poor performance of the maize planted in that area due to a disease pressure during the previous cropping season which resulted in low growth and subsequent excess of soil N available in some areas of the field. This highlights the importance of recording crop management history, especially in managed low N, as it can introduce additional spatial field variability. According to these results (Figures 1b, c, 2B; Table 1), the UAP proved capable of capturing field variability which is extremely useful for any crop improvement program. This is in agreement with the results reported by Verhulst et al. [26] who demonstrated that the intrinsic spatial soil characteristics could be associated to crop performance, and that remote sensing technology could be used to identify areas of low productivity. The authors consistently demonstrated that plots of maize managed as no-till with residue removal had significantly lower mean and minimum NDVI values compared to plots managed as conventional tillage. Considering that the images are geo-referenced, the reflectance data can be used either to design trial layouts which do not include the highly variable portions of the field or in case of harvested trials, to use them as covariate when analyzing the data [23]. In addition, these data can be used to remove spatial variation that is not effectively controlled by blocking. Another option using these data would be to develop management zones based on inherent characteristics of a location [29]. Because this platform can allow covering large areas in a short period of time (compared to manual data collection methods), data can be generated every season and compared to assess changes over time or visualize the impact of any management measure taken to deal with the spatial variability. The ability to closely monitor spatial variability will improve the quality of data collected in trials, which will increase the efficiency of the breeding programs.

**Table 1:** Descriptive statistics for the NDVI values at CIMMYT’s Harare low-N fields, Harare, Zimbabwe

Field	Mean	Min	Max	SD	CV (%)
2	0.3431	0.0243	0.7833	0.1060	26.91
1	0.3234	0.1201	0.4687	0.0321	6.75

*Min* minimum value, *Max* maximum value, *SD* standard deviation, *CV* coefficient of variation (%).

### Plant stress detection

Nitrogen status of field crops can be assessed using leaf or canopy spectral reflectance data [6, 30]. Several studies have found that nondestructive measurements of leaf or canopy reflectance can be used for detecting N-deficient stress in maize [31, 32], rice [33], and wheat [34]. Using aerial multispectral imaging with a UAP, we evaluated the capabilities for remotely sensing low-N stress in maize hybrids. NDVI data generated from multispectral imaging were used to compute a low-N stress index.

**Figure 3:**
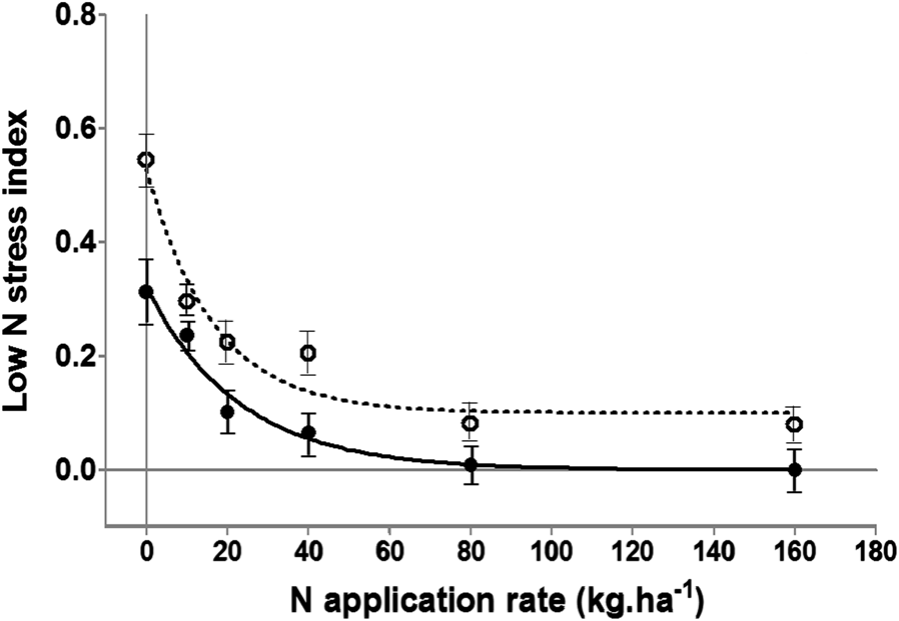
Variation of low-N stress index at 6 N application rates in 3 tolerant hybrids (*solid line*) and 3 sensitive hybrids (*dashed line*). Plants were grown on an N-depleted field at CIMMYT-Harare and data were collected at flowering.

**Figure 4:**
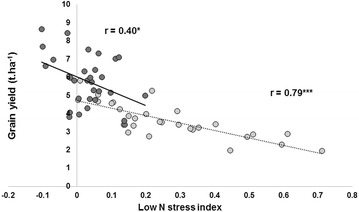
Relationship between grain yield and low-N stress index. Light gray circles are for data from 0 to 40 N rates and dark gray circles from 40 to 160 N rates. Replicated data from 10 hybrids were used (*P ≤ 0.05, ***P ≤ 0.001).

The stress index values decreased from 0 AN to 160 AN (Figure 3). Data showed that this index clearly discriminated between the sensitive and tolerant hybrids. At all the N levels, the sensitive hybrids presented a higher index than the tolerant ones, with the greatest difference when no N was applied (Figure 3). This indicates the higher N requirement of the sensitive genotypes, most probably because of lower N-uptake efficiency. In addition, this index showed a good correlation with grain yield, particularly at low N levels (0, 10 and 20 kg ha^−1^ AN) (Figure 4, r = 0.79, p < 0.001). This is because maize N status is usually significantly correlated with leaf reflectance at low leaf N concentration under field conditions [35] partly due to the fact at low N canopy biomass does not saturate NDVI and therefore the vegetation index remains precise enough.

Our data have shown that remote sensing using a UAP can be used to detect subtle differences of N stress within a field to a resolution of a single row and yet evaluate an entire field (Figure 2). This level of resolution together with the fast data collection that the platform allow, open an avenue for in-season descriptive analysis of plant development variables. With more exploration, this will permit determination of the critical growth stage to consider, the appropriate spectral bands for crop performance analysis, and the integration of co-variate in statistical designs or even the integration of data in crop models.

### Comparison between ground-based measurements and UAV-based remote sensing

#### NDVI

**Figure 5:**
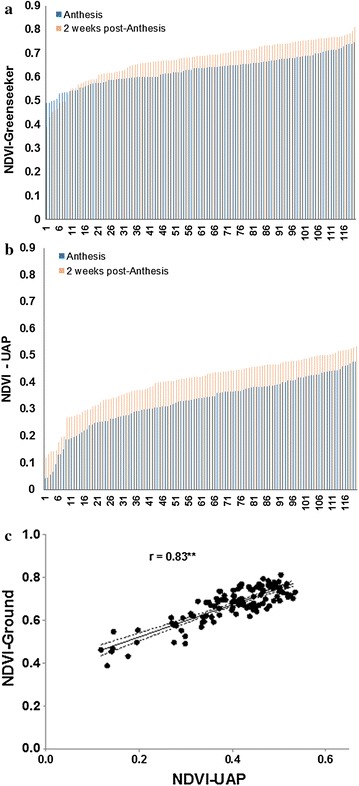
Distribution of NDVI data collected using a ground-based spectroradiometer (**a**) and the UAP (**b**) at two different dates. **c** Correlation between NDVI-Ground and NDVI-UAP. The* dashed lines* represent the 95% confidence intervals (**P ≤ 0.01).

We compared the ground-measured NDVI data with NDVI data derived from the UAP. Data showed that the ground-measured NDVI ranged from 0.5 to 0.65 and 0.4 to 0.8 at flowering and 2 weeks post-flowering, therefore giving an amplitude of 0.15 and 0.2 respectively (Figure 5a, b). The UAP data although lower compared to the ground-measured NDVI gives a higher amplitude of variation 0.35–0.4 at the two measurement time points. In addition there was a good correlation between the two as shown on Figure 5c (r = 0.83, p < 0.001).

#### Crop senescence

Leaf senescence affects the plant’s ability to fill the grains by reducing the grain filling duration. Crop senescence can be used to indirectly assess the ability of a genotype to maintain a higher plant photosynthetic capacity under N deficiency conditions.

**Figure 6:**
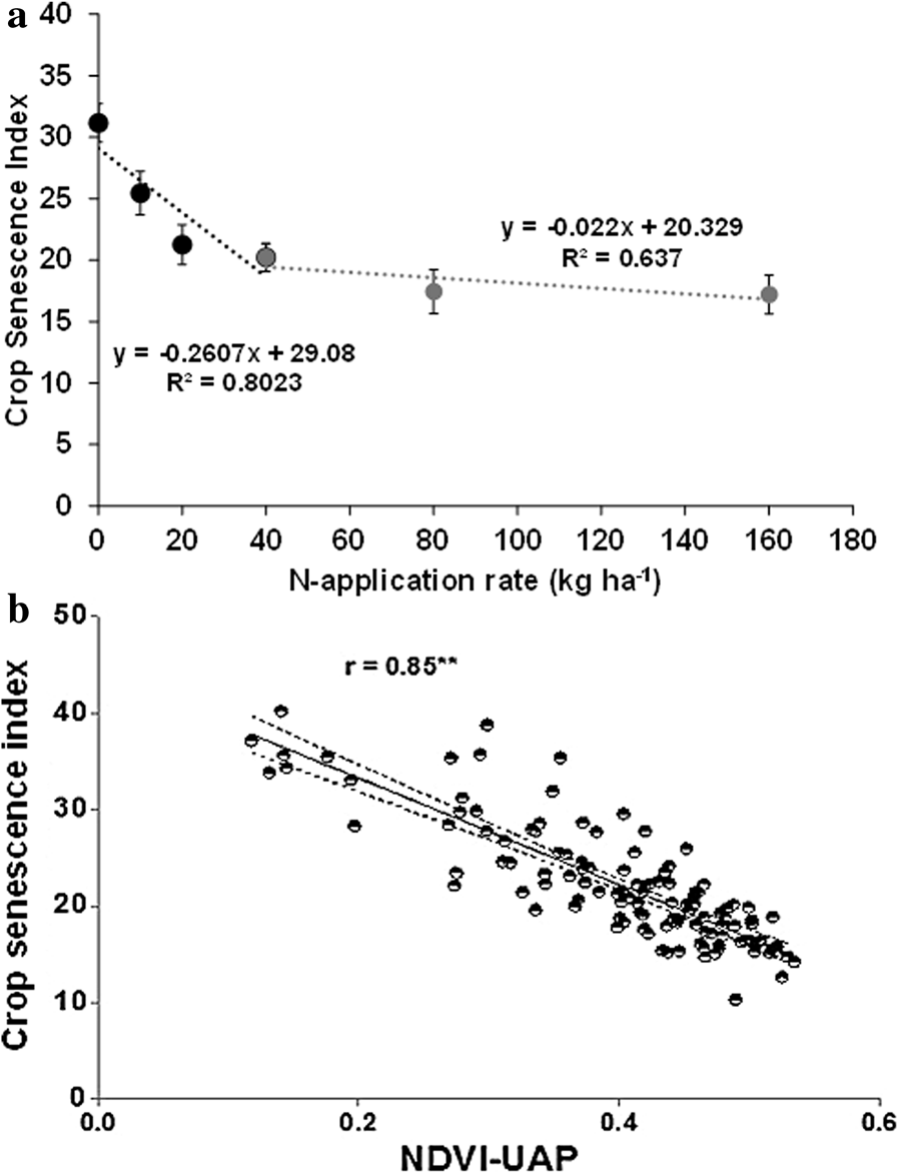
**a** Variation of crop senescence under various N rates and **b** relationship with NDVI extracted from multispectral images taken with the UAP. Replicated data from 10 hybrids and 6 N-application rates were used (**P ≤ 0.01). The *dashed lines* represent the 95% confidence intervals.

Crop senescence index, here formulated from the combination of GA and GAA indices derived from RGB images, presented a large variation and discriminated between the tolerant and susceptible genotypes. Leaf senescence values decreased from 0 kg ha^−1^ N to 160 kg ha^−1^ AN following a broken-stick-model (Figure 6a). Between 0 kg ha-1 N and 40 kg ha^−1^ N, the senescence decrease following a slope of 26% while beyond 40 N, the variation of senescence was small with a slope of only 2.2%. Stress conditions are known to lead to premature senescence. The rapid reduction of senescence between 0 and 40 kg ha^−1^ N is the result of stress reduction due to N supply. Above 40 kg ha^−1^ N the level of N stress is less severe and N application will not result in a significant stress reduction as assessed on leaves. The crop senescence index showed a good correlation with NDVI derived from spectral imaging from the UAP and grain yield (Figure 6b). This underlines that it is possible to use UAP derived spectral imaging to assess leaf senescence in maize plants. Several differences between vegetative and reproductive growth might influence the induction and development of leaf senescence: first, although leaf senescence might be induced by N shortage under field conditions, the timing of N shortage is dependent upon different factors. In the field, the exploration of N sources in deeper soil layers might play the most important role for N uptake during reproductive growth [36].

In addition the variation of the leaf senescence index in relation to N rates show that at low N range, the slope is ten times higher than at high N rates and the relationship is stronger.

Plant leaf senescence decreased with increase in N application rate (Figure 6a). As a consequence of the delay in senescence plant photosynthetic capacity prolongs the effective leaf area duration [37]. On the opposite side, N deficiency accelerates leaf senescence rates throughout the life cycle [38]. This has been shown to be important for ear and kernel initiation, contributing to define maize sink capacity [39] and maintaining functional kernels throughout grain filling with positive impact on the number of developed kernels and kernel final size [40]. Under low soil N conditions, the photosynthetic capacity is reduced due to early senescence as compared to high soil N. The crop senescence index was 20% smaller at 160 kg ha^−1^ AN relative to 0 kg ha^−1^ AN. It was reported that leaf senescence could explain 47% of genotypic variation in nitrogen use efficiency in the field experiments [41].

### Relationship with grain yield

Yield data showed large genetic variation between the 10 hybrids within and among the N treatments. Grain yield ranged from 1.94 to 8.63 t ha^−1^ at 0 and 160 kg ha^−1^ N, respectively (Table 2). At 0 kg ha^−1^ AN application, the yield of low N susceptible hybrids was more than 1.4 t less than that of the tolerant ones. Besides, the yield differences were larger at low N application rates. This is mostly because the differences in growth are more pronounced when N is less available to plants. The strong relationship between the low-N stress index and grain yield (Figure 4) suggest that the genotypic yield differences were partly due to differences in senescence, with genotypes having higher yield showing less senescence as compared to those with lower yield. As reported in many studies, there was a significant negative correlation between grain yield and senescence [42, 43]. Overall, there was a good correlation between NDVI and grain yield (Table 3). The correlation was stronger under low N conditions (0–10 kg ha^−1^) as compared to sufficient N conditions (Table 3). The same trend was observed at flowering and 2 weeks after flowering. Previous works have shown an association between NDVI values and crop biomass accumulation, leaf area index, leaf chlorophyll levels, and photosynthetically active radiation absorbed by the canopy [20, 44], to a large extent because N uptake and NDVI are highly correlated [45]. This has in turn been associated with crop yield [20, 46]. However, this association varies significantly depending on the developmental stage and growth conditions. In most instances, when the leaf area index reaches high values, the association becomes weaker because of saturation effect on NDVI. These studies demonstrate that many factors can potentially affect the detection of genetic variation for vegetation indices, especially with remote sensing methods. These factors include the type of stress tolerance that is being investigated. Moreover the stage of growth to obtain the desired genetic variation would differ whether the data are collected in a low N stress trial like in the present study or under other different stress conditions, such as for example early (planting and emergence) or terminal (i.e. during reproductive stage) drought stress trial.

**Table 2:** Descriptive statistics for the Grain yield values (t ha^−1^) in 10 maize hybrids grown under 6 N application rates

	N-application rate (Kg ha^−1^ AN)					
	0	10	20	40	80	160
Minimum	1.940	2.741	3.373	3.372	5.133	5.284
Maximum	3.896	4.602	5.818	4.998	7.530	8.630
Mean	2.801	3.538	4.654	4.240	6.274	6.821
Std. error	0.129	0.114	0.139	0.120	0.176	0.240

**Table 3:** Coefficients of correlation between grain yield (GY) and (1) NDVI extracted from multispectral images taken with the UAV-platform (NDVI-UAP) and (2) leaf senescence index

	NDVI-UAP	Crop senescence index
All N application rates	0.63***	−0.74***
0 and 10 kg ha^−1^ AN	0.72***	−0.74***
80 and 160 kg ha^−1^ AN	NS	−0.44**

NS: P > 0.05, * P ≤ 0.05, ** P ≤ 0.01, *** P ≤ 0.001.

Regarding crop senescence index, the correlation with grain yield followed the same trend as NDVI (Table 3). The correlation was stronger under low N conditions (0–10 kg.ha^−1^) as compared to sufficient N conditions. N deficiency is known to accelerate leaf senescence rate [38] which was shown to be important for ear and kernel initiation, contributing to define maize sink capacity [39] and maintaining functional kernels throughout grain filling [40].

## Conclusions

The use of UAP for field spatial variability and field-based crop phenotyping is novel, but is expected to become an important tool for improving efficiency in crop breeding. Currently, most of the limitations in the deployment of these platforms in breeding are related to the cost of sensors, spatial resolution of imagery, data processing, management and complexity of operation. The results of the current study suggest that remote sensing from UAP has a great potential for field and crop trait characterization under field conditions. To date, only few studies have been carried out attempting to use UAP’s remote sensing for spatial field variation assessment and crop phenotyping in the field. Our results showed that, this type of platform can be used in low N stress detection/senescence as well as for estimating final yield in maize. However, to effectively deploy this type of platform in a breeding program, there is need to measure its breeding value through selection indices and to have a well-designed data processing and management plan.

## Methodology

### Experimental set up

To address the relevance of UAV-based remote sensing platforms for field crop phenotyping, we set up two experiments at the CIMMYT-Harare research station (−17.725787 S, 31.016457 E) on 5-years nitrogen depleted fields referred here as managed low-N fields. The average Nitrate–N concentration of the managed low-N fields (using the spectrophotometric method) was 8 mg kg^−1^ soil. The first experiment focused on the characterization of the field site for spatial variability. The field was sown with a single wheat variety (*Triticum aestivum* L. cv. SC-Stallion). The planting was done during the winter season 2012 under controlled irrigation to ensure that germination and growth were homogenous. The second experiment focused on assessing N-response of maize (*Zea mays* L.) hybrids. A total of 10 hybrids were used, 5 hybrids were classified as low-N tolerant and 5 hybrids as low-N susceptible based on previous multi-location experiments. Six N fertilization levels were used: 0, 10, 20, 40, 80 and 160 kg ha^−1^ of Ammonium Nitrate (AN). Except in the case of 80 and 160 kg ha^−1^ of AN, where a split application was used (50% at knee-high and 50% prior to anthesis), N fertilization was performed at knee-high stage. The experiment was a split-plot design with hybrids as main plots and N levels as subplots, replicated three times in a randomized complete block design. The experiment was planted on the 23rd December 2013 as 2 rows per plot. The rows were 75 cm apart and 4 m long with 17 planting stations per row. To reduce border effects, 2-row plots of a commercial variety were planted on all sides of each treatment. Single super phosphate fertilizer (14% P_2_O_5_: 7% K_2_O) was applied to all the plots at a rate of 400 kg ha^−1^, to supply the crop with phosphorous and potassium. Hand weeding was used to control weeds.

### Unmanned aerial platform

**Figure 7:**
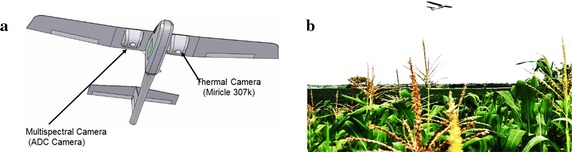
**a** Fixed-wing UAV-based remote sensing platform (UAP) equipped with ADC-Lite, Tetracam camera and a Miricle thermal camera (not used in this study). **b** The UAP flying over a maize field.

The experiment was conducted using an unmanned aerial vehicle-based remote sensing platform (Figure 7) developed by Airelectronics (Madrid, Spain) in collaboration with the University of Barcelona, the QuantaLab at the Institute for Sustainable Agriculture (IAS-CSIC), the Crop Breeding Institute-Zimbabwe and CIMMYT. The UAP is a fixed-wing platform controlled by an autopilot system that enables autonomous navigation, based on coordinates set in the flight plan designed with U-See 1.190 software (Airelectronics, Madrid, Spain). It has an automatic GPS waypoint navigation and altitude control.

This platform has the capability of flying at a wide range of altitudes and is capable of carrying a payload of up to 1.5 kg and flying for 30 min. It can cover up to 40 ha for a 30 min flight at an altitude of 150 m above ground level.

### Imagery acquisition and data collection

#### UAP

Images were collected from the wheat experiment to assess spatial variability 1 week prior to booting and for the whole N-response trial on two dates (anthesis and 2 weeks after anthesis). They were acquired using the ADC-Lite multispectral camera mounted on the UAV platform near midday under cloud-free conditions. The ADC-Lite is a specialized light weight agricultural multispectral camera (ADC-Lite, Tetracam, Inc., Chatsworth, CA, USA) with a 3.2 megapixel Complementary Metal Oxide Semiconductor (CMOS) sensor. The camera’s sensor has a ground resolution of 60 mm per pixel and 123 × 92 m Field of View at an altitude of 150 m above ground level. The camera is optimized to simultaneously capture Green, Red and NIR channels with bands approximately equal to Landsat Thematic Mapper TM2 (520–600 nm), TM3 (630–690 nm) and TM4 (760–900 nm) and has a lens focal length of 8.0 mm. The UAP followed a flight plan made of 6 waypoints covering the entire field at an altitude of 150 m above terrain and a velocity of 45 km h^−1^. The images (2048 × 1536 pixels) were taken at the rate of one every 5 s.

#### Hand-held instruments

Different ground-based sensors were used to collect data. A leaf chlorophyll meter (Minolta SPAD-502, Spectrum Technologies Inc, Plainfield, IL, USA) a spectroradiometer provided with an active sensor (GreenSeeker handheld crop sensor, Trimble, USA) as well as a conventional digital camera (Cyber-shot DSC-WX80, Sony, Japan) were used to measure spectroradiometrical leaf canopy (NDVI) and RGB (red/green/blue) image derived vegetation indices, respectively, as detailed in the data procession section.

### Image data processing

#### Color infra-red multispectral images

A radiometric calibration was performed after applying a channel decomposition, which consists of demosaicing the infrared color filter array (CFA) to reconstruct each G-R-NIR sample from the undersampled ones. The imagery was synchronized through the GPS position and triggering time recorded for each image, without the usage of any additional inertial units. For this study, only absolute positions were used to generate the ortho-rectified mosaics, following by the steps of image registration, calibration and blending.

The multispectral images acquired by the UAP enabled identification of each individual row (Figure 2) and the extraction of maize plant reflectance values. For each row, a region of interest (ROI) was established manually in the center of the row based on visual detection of the edges/soil to extract reflectance values (Figure 2a) using only vegetation pixels. The image reflectance data extracted from each treatment field, coinciding with each flight time, were subsequently used to compute the vegetation indices used in the analysis.

The Normalized Difference Vegetation Index, NDVI = (R800 − R670)/(R800 + R670), [45] was calculated from the imagery and compared with the ground-measured NDVI, then used to compute a nitrogen stress index to assess whether the effects of low-N stress on maize could be captured effectively by the platform. The nitrogen stress index was calculated as:

$${\text{NSI}}_{{({\text{NDVI}})}} = 1 - {\text{NDVI}}_{\text{i}} /{\text{NDVI}}_{\text{m}}$$

where NDVI_i_ is the NDVI value at a given N application rate i and NDVI_m_ the average NDVI value at 160 kg ha^−1^ (used here as a reference).

#### RGB images

The RGB images from the digital camera were analyzed using the open source Breedpix 0.2 software designed for the digital photographs processing. This software enabled the extraction of RGB vegetation indices in relation to different properties of color [47]. The procedures for calculating the vegetation indices are described in [48]. Basically, the green fraction (GF), corresponds to the proportion of green pixels in an image, where a pixel is considered green if its hue is within the range 60–180°. The greener fraction (GGF) was aimed at quantifying the fraction of fully functional green cover, excluding yellowish pixels that correspond to senescent leaves, and was calculated as the proportion of pixels whose hue is within the range 80–180°. Image analysis was used to define an index of crop senescence:

$${\text{Crop senescence index}} = 100 \times \left( {{\text{CGF}} - {\text{CGGF}}} \right)/{\text{CGF}}$$

where CGF is the crop green fraction and CGGF the crop greener fraction.

### Statistical analysis

The NDVI and yield data were subjected to classical statistical analysis to obtain (1) descriptive statistics mean, minimum, maximum, standard deviation and coefficient of variation and (2) correlation coefficients using GraphPad Prism (GraphPad Software Inc., San Diego, CA, USA, 1996).
